# Millets for little ones: child feeding practices and nutritional profile of millet-based foods in Telangana, India

**DOI:** 10.3389/fnut.2025.1648217

**Published:** 2025-10-08

**Authors:** Ilene Maria Shaji, Shreyas Patil, Remya Mary John, Abhirami Renjith, Chandralekha Kona, Mounika Reddy, Gomathi Ramaswamy

**Affiliations:** ^1^All India Institute of Medical Sciences, Bibinagar, Telangana, India; ^2^Department of Community Medicine and Family Medicine, All India Institute of Medical Sciences, Bibinagar, Telangana, India; ^3^Department of Pediatrics, All India Institute of Medical Sciences, Bibinagar, Telangana, India

**Keywords:** millet, malnutrition, under-5 children, packaged food, RTE and RTC

## Abstract

**Background:**

Millets are nutrient-dense, climate-resilient grains with the potential to combat child malnutrition. There is limited data available on millet consumption among under-five children and the nutritional quality of millet-based ready-to-eat (RTE) and ready-to-cook (RTC) products available in the market, specifically for under-five children in India.

**Methods:**

The study methodology has two components: (i) A cross-sectional study was conducted in Telangana, India, among 384 mother–child dyads attending a tertiary care hospital. Data on millet-related feeding practices were collected from the mothers through interviews. (ii) Nutrient profiles and labeling details of millet-based RTE and RTC products were assessed by visiting supermarkets, bakeries, and online grocery markets in two districts of Telangana.

**Results:**

While 99% of mothers were aware of millets, only 60% included them in their child’s diet at least for 2–3 days a week. Children who consumed millets showed significantly higher height, weight, and MUAC compared to non-millet-consumed children (*p* < 0.05). Most millet-based RTE and RTC products met energy, protein and iron needs, but had low calcium content as per the estimated average requirement (EAR) for 100 g of the food across age groups. The sodium content of the 20% RTC millet foods was sufficient to fulfill 75% of the recommended daily allowance of children aged 6 months to 6 years. All food product labels had information on ingredients, manufacturing, expiry dates, and lot/ batch numbers. However, there were lacunae in mentioning the additives/food color, suitable age range for consumption, storage conditions and allergen information.

**Conclusion:**

Mothers of under-five children in Telangana India have good knowledge of millets. However, 40% of them did not provide millet to the children regularly. The children who consumed millets had better anthropometric indicators compared to those who did not consume. There is a need to strengthen the food labeling practices on RTC and RTE millet-based foods.

## 1 Introduction

Childhood malnutrition is a major public health concern, with about 45% of under-five children’s deaths being linked to undernutrition (1). According to the WHO-UNICEF-World Bank joint malnutrition estimates, more than one in five (148.1 million) under-five children were stunted, and 45 million were wasted in 2022 (2). India’s National Family Health Survey (NFHS-5), 2019–2021, reported that about 35.5% of under-five children in the country are stunted, 7.7% are severely wasted, 32.1% are underweight, and 3.4% are overweight (3). There is also a rising concern about over nutrition and micronutrient deficiencies, which contribute to the triple burden of malnutrition along with under nutrition among children. To combat this, India has implemented programs such as the Infant and Young Child Feeding (IYCF) program, Integrated Child Development Services (ICDS), Poshan Abhiyaan, and the Anemia Mukt Bharat program (4, 5). In addition to these centrally sponsored programs, states have also implemented context-specific programs to address the triple burden of malnutrition. Despite these efforts, the burden of malnutrition remains high in India.

Millets are one of the oldest known and highly nutritious crops, grown widely across semi-arid and tropical regions, mainly in Asia and Africa. They are dubbed “smart foods,” with numerous nutritional and health benefits. They also have environmental benefits as they are highly water-efficient crops with a low carbon footprint (6). Their climate-resilient nature and ability to survive in high temperatures and low water conditions benefit farmers. They are also called “nutri-cereals” due to their rich content of macronutrients and micronutrients like B-vitamins, iron, calcium, potassium, zinc, magnesium, and dietary fiber (7, 8). Certain groups of millets, such as pearl millets (Bajra), are gluten-free and dietary fiber-rich and can be consumed by individuals with celiac disease, constipation, and those at risk of developing gallstones. Bioactive compounds derived from millets have been found to have antioxidant, antihypertensive, ACE-inhibitory, antiproliferative/anticancer, antidiabetic, antimicrobial, anti-inflammatory, antifungal, and anti-coagulant effects (9). Millets could therefore be beneficial in the management of chronic diseases such as obesity, cardiovascular diseases, cancer, diabetes, as well as asthma and migraine (7, 10). As a result of these properties, millets have the potential to address different forms of malnutrition while ensuring dietary diversity (11). Moreover, these resilient “nutri-cereals” or “smart foods” can play a major role in fulfilling the Sustainable Development Goal (SDG 2) toward eliminating hunger and all forms of malnutrition by 2030 (12).

India is the largest producer of millets, accounting for 80% of the millet production in Asia and 20% of the global production (13). The country cultivates both major millets (larger in size) like Sorghum (Jowar), Pearl Millet (Bajra), and Finger Millet (Ragi), and minor millets (smaller in size) like Little millet, Proso millet, Kodo millet, Foxtail millet, Barnyard millet, and Buckwheat millet. Within the country, Rajasthan state is the top-most contributor to the country’s total millet production, followed by Maharashtra, Uttar Pradesh, Karnataka, and Madhya Pradesh. In 2023, India produced around 16 million tonnes of millet, with Bajra (9.7 million tonnes) being the leading millet produced, followed by Jowar (4.1 million tonnes), Ragi (1.7 million tonnes), and minor millets (0.3 million tonnes) (14). India has also taken a step to make millets available for all citizens by supplying them through various programs such as Targeted Public Distribution System (TPDS), Pradhan Mantri Poshan Shakti Nirman (PM POSHAN), Integrated Child Development Services (ICDS), National Food Security Mission (NFSM), and Other Welfare Schemes (OWS).

Acknowledging the various nutritional and health benefits of millets, the National Institute of Nutrition, Indian Council for Medical Research (NIN-ICMR) recommends that at least half of the recommended cereals be whole grain millets (“My Plate for day”). For children aged below 10 years, 20% by raw weight of cereals should be from millets, and it’s one of the five complementary food items recommended for minimum dietary diversity among infants and children. Millets like foxtail millet are rich in calcium and are recommended for children and adolescents for attaining optimal peak bone mass (15). Recent technologies have brought millet products with enhanced taste, nutritional quality, improved shelf-life, and convenience. These products are currently available as millet-based Ready-To-Eat (RTE) foods like snacks (puffs, chips, khakhra), instant food (flakes), bakery products (bread, biscuits, cookies), and millet-based Ready-To-Cook (RTC) foods such as instant foods (noodles, pasta, pizza base, semolina, vermicelli), mixes (idli, upma, dosa, porridge), and beverages (malt drinks, millet-plus-milk-based beverages). Some of these are fortified with micronutrients such as iron, vitamins, zinc, etc. (16–18). These RTE and RTC products, especially those designed for children, are available in attractive packages to improve uptake and compliance (19). Studies from India among adolescents (10–14 years) and children (3–6 years) reported better improvement in anthropometric parameters while consuming millet-based diet regularly compared to a non-millet-based based (cereal based) diet (20, 21). A systematic review from India reported that millets are effective in improving the hemoglobin levels by increasing the bioavailability of iron on regular consumption of millets, indicating the efficacy of millets on micronutrient deficiencies as well (22). However, our literature search revealed that the utilization of millet-based foods by mothers of under-five children and the nutrient profile of the millet-based products available in the Indian market have not been explored. Hence, this study assessed the millet-based dietary practices followed among mothers of under-five children attending a tertiary care hospital in Telangana, India. The study also describes the nutrient profile and packaging of the millet-based products available in the market for under-five children in India.

## 2 Methodology

The study was a cross-sectional study conducted over 4 months from October 2023 to January 2024 in the Yadadri Bhuvanagiri and Hyderabad districts of Telangana, India. Telangana, a newly formed Indian state in 2014, has a population of 3.5 crores spread across 33 administrative units - districts. Telangana ranks 7th in terms of millet production in the country, with Sorghum being the major millet produced, followed by Ragi, Bajra, and other minor millets. However, Paddy remains the largest produced and also Telangana’s staple food. Yadadri Bhuvanagiri district is one among the 33 districts of Telangana, with 17 mandals and revenue districts. The district has a tertiary care center, All India Institute of Medical Sciences (AIIMS), Bibinagar, providing various multispecialty health services catering primarily to Yadadri Bhuvanagiri and other surrounding districts of the state. The pediatric health services at the center predominantly serve the children directly from the community and the children referred by primary or secondary health centers. The millet-related dietary practices of the under-five children were assessed among the mothers of children visiting the pediatrics outpatient department of the tertiary care center. The nutrient profile of millet-based products was evaluated in two adjacent districts - Yadadri Bhuvanagiri and Hyderabad- and was also included among the products available on mainstream online platforms. The Hyderabad district is also one of the largest metropolitan cities of India.

### 2.1 Millet consumption practices among under-five children

Mothers of children aged 6–59 months visiting the Pediatrics Outpatient Department (OPD) at AIIMS Bibinagar, Telangana, were approached for consent and requested to share their child feeding practices. Mothers of children with severe illness, those requiring inpatient admission, children with malabsorption syndromes, congenital anomalies, and developmental delays were excluded as their anthropometric parameters would have been affected by the illness or their long-term health conditions. Assuming that at least 50% of the mothers would provide millet-based food in any form for their under-five children, with 5% absolute precision and 95% confidence interval, the sample size was calculated as 384. The footfall of the pediatric OPD during the study period was 80–100 per day, and the OPD services are available from Monday to Saturday from 9 a.m. to 1 p.m. Systematic random sampling with a class interval of five was used to enroll the participants. The class interval was decided based on the previous OPD footfall, and the children were enrolled based on the sequence they were registered in the pediatric OPD for consultation and their age category. If the eligible child identified from the register was not willing to participate in the study next eligible child was approached to participate in the study. A pre-tested, semi-structured questionnaire was employed to collect information among mothers of under-five children regarding socio-demographic details like age, gender, mother’s education, occupation, knowledge, and existing practices on including millets in the daily diet of children, feeding frequency, and formulation of millet food given to children. Data for practices related to millet inclusion in children’s diet were collected with respect to the last 1-month period to reduce recall bias. The children’s anthropometric parameters (height, weight, and mid-upper arm circumference) were measured per WHO guidelines.

### 2.2 Nutrient content and product profile

Online and physical market surveys were conducted to identify millet-based products available in bakeries and grocery stores for children’s consumption, and their composition, nutritional content, and fortification details were obtained from the food label available on the package. The online survey was done by browsing food and grocery delivery applications such as Big Basket, Amazon, Flipkart, and First Cry, which have their services in and around Hyderabad and Yadadri Bhuvanagiri districts. For the offline part of the survey, we visited eight supermarkets (each had a footfall of at least 300 in a day) and five bakeries from five randomly selected mandals of Hyderabad and Yadadri Bhuvanagiri districts.

A structured checklist was used to capture information from the product packaging, which included the selling format (RTE/RTC), age specification for use among children, presence of millet, number of millets added in the product, ingredients label, nutrition information per feed, sugar/salt content, cooking instructions, FSSAI approval, fortification details, packaging quality, and the product packing attractiveness. The last two parameters were collected as an observation by the investigator using a Likert scale.

### 2.3 Data entry and analysis

Two investigators, IMS and AR, collected the data for the study. The data from both surveys were captured in paper format, then entered into the Epicollect5 mobile application, and analyzed using Stata version 18 software (StataCorp, 2023, College Station, TX: StataCorp LLC). The continuous variables like age, height, weight, and mid-arm circumference were summarized as mean ± SD or median (IQR). The categorical variables, like gender, dietary diversity, nutritional contents of millet products, fortification, and labeling practices, were summarized as frequencies and percentages. The nutritional status of children consuming/ not-consuming millet-based food was compared using an unpaired t-test or the Mann–Whitney U test for height, weight, and mid-arm circumference, and the chi-square test of association for classification based on weight-for-height/length (WFH/WFL). The nutrient profile of the millet-based products was summarized using percentages. We also tried to assess the quantity of energy, protein, sodium, calcium, and iron provided by the millet-based RTC and RTE food items. The percentage of energy, protein, sodium, calcium, and iron available from 100 grams of the RTC/ RTE item was calculated against the estimated average requirement (EAR) and recommended daily allowance (RDA) guidelines of the ICMR-National Institute of Nutrition, India guidelines, 2024 for 6 months to 1 year, 1 year to 3 years, and 3 years to 6 years of children as categorized in this guideline (15). The percentage of nutrition availability was expressed as <25%, 26–50%, 51–75, and>75% of EAR/RDA per 100 grams of RTC/RTE food.

### 2.4 Ethical clearance

Approval from the Institute Ethics Committee (AIIMS/BBN/IEC/SEPT/2023/304), AIIMS Bibinagar, was obtained before starting the study. Written informed consent was obtained from the mothers of under-five children who participated in the study.

## 3 Results

A total of 384 mother–child dyads participated in the study. The mean (SD) age of mothers was 25.8 (3.4) years, and 97.1% of them were the primary caretakers of their children. The majority of mothers (51%) had completed high school (10 years of formal schooling) or intermediate (12 years of formal education), while another 40.4% of the mothers had attained graduation and post-graduation. The majority of the mothers interviewed were homemakers (91.9%), and the median (IQR) income of the families was Rs. 25,000 (10000). The mean (SD) age of the children was 24.3 (14.7) months, 57.6% were male, and 57.3% belonged to nuclear families. (Table 1).

**Table 1:** Socio-demography and characteristics of the study participants (*n* = 384).

Participant characteristics (*n =* 384)	N	(%)
Age of under five children (in months) mean (SD)	24.3	(14.7)
Age of mother (in years) mean (SD)	25.8	(3.4)
Education status of the mother		
No formal education	7	(1.8)
Primary/middle school	26	(6.8)
High school/Intermediate	196	(51)
Graduate and above	155	(40.4)
Occupation of the mother		
Homemaker	353	(91.9)
Unskilled/semiskilled	3	(0.8)
Skilled worker	10	(2.6)
Clerical/shop/farm	3	(0.8)
Semiprofessional	10	(2.6)
Professional	5	(1.3)
Monthly family income in INR-median (Q1, Q3)	20,000	(15,000, 30,000)
Gender of child		
Male	221	(57.6)
Female	163	(42.4)
Type of family		
Nuclear	220	(57.3)
Joint	36	(9.4)
Three-generational	127	(33)
Broken	1	(0.3)
Usual caretaker of the child at home:		
Mother	373	(97.1)
Grandmother	9	(2.4)
Housemaid/helper	2	(0.5)
Perceptions & practices among mothers related to millet foods		
Only aware about millets (*n* = 384)	381	(99.2)
Mothers who include millet in their child’s diet (*n* = 381)	190	(49.9)
Mothers who provide more than one millet to their child (*n* = 190)	87	(45.8)
Source of knowledge of mothers about the importance of millet foods (*n* = 190)		
Family/friends	123	(64.8)
Asha/Anganwadi workers	28	(14.7)
Healthcare workers	13	(6.8)
Audio-visual media-television, social media, radio	19	(10)
Print media-newspapers, books	7	(3.7)
Frequency of feeding millet food to child (*n* = 190)		
Everyday	68	(35.8)
2–3 days in a week	47	(24.7)
Once a week	37	(19.5)
Not sure	38	(20.0)
Form in which millet food is given (*n* = 190)^*^		
As major meal	137	(72.1)
As snack	19	(10.0)
As drink	83	(43.7)
Liking of child toward millet food (*n* = 190)		
Likes very much	31	(16.3)
Likes to a good extent	94	(49.5)
Neutral	39	(20.5)
Does not like very much	17	(9.0)
Does not like at all/have to force	9	(4.7)
Anthropometry of child		
Height (in cm) mean (SD)	82	(12.6)
Weight (in kg) mean (SD)	10.2	(4.6)
Mid-upper arm circumference (in cm)-mean (SD)	15.2	(1.4)
Weight-for-height/length (WFH/WFL)		
Normal (-2SD to +2SD)	256	(66.7)
Moderate acute malnutrition (MAM) [-2SD to -3SD]	74	(19.3)
Severe acute malnutrition (SAM) [< -3SD]	54	(14.1)

*Multiple response questions.

### 3.1 Dietary practices among under-five children

Almost all mothers (99.2%) were aware of millets, with most being aware of jowar and ragi (99%), followed by bajra (69.8%). Half of the mothers included millets in their child’s diet. Around 45.7% provided more than one millet to their child. Ragi (85.8%) was the most commonly included millet in the children’s diet, followed by jowar (58.4%), bajra (5.8%) and foxtail millet (5.8%). Among the children who were consuming millets (*n* = 190), only 35.8% had millets as part of their daily diet; 24.7% consumed it 2–3 times a week, whereas around 20% consumed it once weekly. Among these 190 children, 72.1% of them consumed millets as a major meal (roti, porridge, dosa, idli, khichdi, etc) which was prepared from a single or multiple type of millets, 43.7% had millets in the form of a drink and 10% had it as a snack (ladoos, biscuit, etc.). Around 4% mothers used packaged millet food products, out of which RTE foods, such as biscuits, and RTC foods, such as breakfast cereals, were most commonly reported. The mothers of these 190 children mentioned that 65.8% of the children either liked millet-based food very much or to a good extent, while 4.7% did not like the millet food at all. When asked about the reason for not including millets in their child’s diet, the most common reasons were found to be ‘unaware of millet usage as a diet among children’, ‘millets may cause indigestion’, and ‘child not liking the taste of millets (Table 1).

Analysis of anthropometric data of the children revealed that the overall mean (SD) of height was 82 (12.6) cm, weight was 10.2 (4.6) kg, and mid-upper arm circumference (MUAC) was 15.2 (1.4) cm. On calculating the weight-for-height/ length (WFH/WFL), it was observed that 19% children fell under the moderate acute malnutrition (MAM) category (WFH/WFL between -2SD and -3SD z scores) while 14.1% were found to be in severe acute malnutrition (SAM) category (WFH/WFL < -3SD z score). The mean (SD) heights among children who were fed millets and those who were not were 84.7 (11.0) cm and 80.6 (10.3) cm, and this difference was found to be statistically significant (*p* < 0.001). Similarly, the difference in weights [mean (SD)] of millet-fed [10.6 (2.63) kg] and non-millet-fed [9.48 (2.30) kg] children was also found to be statistically significant, *p* < 0.001. The average MUAC [mean (SD)] of children who were fed millets [15.4 (1.37) cm] was also significantly higher than those who were not fed millets [15.0 (1.31) cm]. Moreover, MAM was observed in 15.3% of millet-fed children compared to 23.2% in those who were not fed millets, while SAM was noted in 11.6% of millet-fed children as opposed to 16.5% in those who were not fed millets. A statistically significant association between WFH/WFL and dietary patterns was detected by the chi-square test (*p* = 0.028; Table 2).

**Table 2:** Anthropometric details of under-5 children based on inclusion of millets in diet (*n* = 384).

Anthropometry	Millet-fed children (*n* = 190)	Non-millet fed children (*n* = 194)	*p*-value
Height in cm (Mean ± SD)	84.7 ± 11.0	80.6 ± 10.3	<0.001*^a^
Weight in kg (Mean ± SD)	10.6 ± 2.63	9.48 ± 2.30	<0.001*^a^
Mid-upper arm circumference in cm (Mean ± SD)	15.4 ± 1.37	15.0 ± 1.31	0.013*^a^
Weight-for-height/length (WFH/WFL) n (%)			
Normal [Z score -2SD to +2SD]	139 (73.2%)	117 (60.3%)	0.028*^b^
MAM [Z score -2SD to -3SD]	29 (15.3%)	45 (23.2%)	
SAM [Z score < -3SD]	22 (11.6%)	32 (16.5%)	

MAM, Moderate acute malnutrition; SAM, Severe acute malnutrition; ^a^unpaired t-test; ^b^Chi-square test. *represent the statistically significant *p* values.

### 3.2 Nutrient profile of millet-based products available in the market

#### 3.2.1 Nutrient content assessment

A total of 290 millet-based food products were identified from the market survey (online and physical survey). About 47.6% of the identified products were in the RTE format, 28.3% were of the instant mix type, i.e., they needed to be mixed with water or milk, while 24.1% were of the RTC variety. More than half (55.5%) of the products had no mention of the age-categories for which the food product was suitable, while most (96.2%) of the products analyzed had taglines related to health claims such as ‘various multigrain and millets included’, ‘diabetic friendly’, ‘natural & healthy’, ‘zero junk promise’, ‘fiber-rich’, ‘tasty and convenient’, ‘gluten-free’, etc. Most of the food products were labeled vegetarian (90%) and the rest were vegan. All products (100%) bore the label that indicated the ingredients present; ragi (56.9%) was the most common millet, followed by jowar (47.2%) and bajra (27.6%). The quantity of millet for every 100 grams of the product was mentioned on the label in 133 (45.9%) products. Nutritional information such as energy (kilocalories), carbohydrate (grams), protein (grams), and fat (grams) per 100 grams of the product was present on all products. However, information related to saturated fats (grams), trans fats (grams), sugar (grams), and fiber (grams) was missing on the label from 30.7, 31.4, 16.6, and 2.4% of the products, respectively.

#### 3.2.2 Nutrient quantity of millet-based foods

Assessment of the nutrient quantity per 100 grams of the RTC and RTE millet-based foods is depicted in Table 3. This table depicts the nutrient quantity that children will receive if they are provided 100 g of RTC or RTE, compared against the Recommended Daily Allowance (RDA) or Estimated Average Requirement (EAR) for the specific age of the child. For the age group of 6 months - 1 year, 88.4% of RTC items had an energy availability of 51–75% of the EAR, while 92.8% of the items had protein availability of more than 75% of the RDA. The availability of sodium in 39% of RTC items was more than 75% of the RDA. Compared with the nutritional requirements of children aged 1–3 years, the energy availability in 89.9% of RTC items was 26–50% of the EAR, 60.9% of the items had a protein availability that was more than 75% of the RDA and iron availability in 34.6% of the items was more than 75% of the RDA. For children aged 3–6 years, 68.1% of the RTC items had an energy availability that was 26–50% of the EAR, and 53.6% of the items had a protein availability ranging from 51 to 75% of the RDA. Calcium content was less than 25% of the RDA in more than 90% of the RTC products for all the age groups.

**Table 3:** Nutritional profile of the millet-based products (100 grams) in terms of estimated average requirement (EAR) and recommended daily allowance (RDA).

Variable	Estimated average requirement (EAR)										Recommended daily allowance (RDA)									
	% availability <25%		% availability 26–50%		% availability 51–75%		% availability >75%		Total		% availability <25%		% availability 26–50%		% availability 51–75%		% availability >75%		Total	
	N	(%)	N	(%)	N	(%)	N	(%)	N	(%)	N	(%)	N	(%)	N	(%)	N	(%)	N	(%)
Ready to cook (RTC) items																				
Energy																				
6months-1 yr		-	8	(11.6)	61	(88.4)		-	69	(100)			-		-		-		-	
1–3 yr	7	(10.1)	62	(89.9)		-		-	69	(100)			-		-		-		-	
3–6 yr	22	(31.9)	47	(68.1)		-		-	69	(100)			-		-		-		-	
Protein																				
6months-1 yr			1	(1.4)			68	(98.6)	69	(100)			1	(1.4)	4	(5.8)	64	(92.8)	69	(100)
1–3 yr			1	(1.4)	8	(11.6)	60	(87)	69	(100)	1	(1.4)	2	(2.9)	24	(34.8)	42	(60.9)	69	(100)
3–6 yr	1	(1.4)	2	(2.9)	26	(37.7)	40	(58)	69	(100)	1	(1.4)	12	(17.4)	37	(53.6)	19	(27.6)	69	(100)
Sodium																				
6months-1 yr											24	(58.5)	1	(2.5)			16	(39)	41	(100)
1–3 yr											25	(61)			7	(17.1)	9	(21.9)	41	(100)
3–6 yr											25	(61)	6	(14.6)	3	(7.3)	7	(17.1)	41	(100)
Calcium																				
6months-1 yr		-		-		-		-		-	21	(91.4)	1	(4.3)	1	(4.3)		-	23	(100)
1–3 yr	22	(95.6)	1	(4.4)		-		-	23	(100)	22	(95.6)	1	(4.4)		-		-	23	(100)
3–6 yr	22	(95.6)	1	(4.4)		-		-	23	(100)	22	(95.6)	1	(4.4)		-		-	23	(100)
Iron																				
6months-1 yr	3	(11.5)			1	(3.9)	22	(84.6)	26	(100)	3	(11.5)	1	(3.9)	3	(11.5)	19	(73.1)	26	(100)
1–3 yr	4	(15.4)	5	(19.2)	4	(15.4)	13	(50)	26	(100)	6	(23.1)	7	(26.9)	4	(15.4)	9	(34.6)	26	(100)
3–6 yr	6	(23.1)	7	(26.9)	4	(15.4)	9	(34.6)	26	(100)	9	(34.6)	8	(30.8)	2	(7.7)	7	(26.9)	26	(100)
Ready to eat (RTE) items																				
Energy																				
6 months-1 yr	10	(4.5)	12	(5.5)	130	(58.8)	69	(31.2)	221	(100)		-		-		-		-		-
1–3 yr	15	(6.8)	187	(84.6)	17	(7.7)	2	(0.9)	221	(100)		-		-		-		-		-
3–6 yr	35	(15.8)	184	(83.3)	2	(0.9)			221	(100)		-		-		-		-		-
Protein																				
6 months-1 yr	6	(2.7)	10	(4.5)	19	(8.6)	186	(84.2)	221	(100)	7	(3.2)	9	(4)	36	(16.3)	169	(76.5)	221	(100)
1–3 yr	7	(3.1)	9	(4.1)	55	(24.9)	150	(67.9)	221	(100)	9	(4.1)	29	(13.1)	83	(37.6)	100	(45.2)	221	(100)
3–6 yr	10	(4.5)	31	(14)	87	(39.4)	93	(42.1)	221	(100)	16	(7.3)	76	(34.4)	75	(33.9)	54	(24.4)	221	(100)
Sodium																				
6 months-1 yr		-		-		-		-		-	64	(54.3)	22	(18.6)	10	(8.5)	22	(18.6)	118	(100)
1–3 yr		-		-		-		-		-	84	(71.2)	16	(13.5)	8	(6.8)	10	(8.5)	118	(100)
3–6 yr		-		-		-		-		-	92	(77.9)	10	(8.5)	12	(10.2)	4	(3.4)	118	(100)
Calcium																				
6 months-1 yr		-		-		-		-		-	31	(36.1)	22	(25.6)	7	(8.1)	26	(30.2)	86	(100)
1–3 yr	41	(47.7)	18	(20.9)	9	(10.5)	18	(20.9)	86	(100)	49	(56.9)	13	(15.1)	12	(14)	12	(14)	86	(100)
3–6 yr	49	(56.9)	13	(15.1)	12	(14)	12	(14)	86	(100)	52	(60.4)	13	(15.1)	12	(14)	9	(10.5)	86	(100)
Iron																				
6 months-1 yr		-		-	5	(6.2)	75	(93.8)	80	(100)		-	5	(6.3)	6	(7.5)	69	(86.2)	80	(100)
1–3 yr	5	(6.2)	14	(17.5)	19	(23.8)	42	(52.5)	80	(100)	9	(11.2)	21	(26.2)	23	(28.8)	27	(33.8)	80	(100)
3–6 yr	9	(11.2)	21	(26.2)	23	(28.8)	27	(33.8)	80	(100)	13	(16.2)	35	(43.8)	19	(23.8)	13	(16.2)	80	(100)

A similar assessment of ready-to-eat (RTE) millet-based foods found that for children aged 6 months −1 year, 31% had an energy availability more than 75% of the EAR. The availability of sodium in 19% of products was more than 75% of RDA, and calcium in 36% of products was less than 25% of the RDA. On the other hand, for iron, the availability in 86.2% of the products was more than 75% of the RDA in this age group. Compared with the nutritional requirements of children aged 1–3 years, the energy availability in 84.6% of items was 26–50% of the EAR. The availability of calcium in 57% of the items was less than 25% of the RDA while the iron availability in 34% of the items was more than 75% of the RDA. For children aged 3–6 years, 68.1% of the items had an energy availability that was 26–50% of the EAR, and 42% items had a protein availability more than 75% of EAR. The availability of sodium in 78% of the items, and calcium in 60% of the items was less than 25% of the RDA while the iron availability in 16% of the items was less than 25% of the RDA.

#### 3.2.3 Packaging and labeling assessment

Information related to additives, flavoring and coloring was present on most (91.7%) products, with 73.8% of the products claiming to have no added flavors and/or colors. Instructions for cooking, excluding RTE foods, were mentioned on 97.2% of the products. These instructions were written in English on all products, and were written in Hindi and Tamil in 2.3 and 1.1% of the products, respectively. Food Safety and Standards Authority of India (FSSAI) label was present on 98.6% of the products while an organic certification was present on 29.6% products. Fortification was mentioned on the packaging of two products, however not much information was given on the details of the fortified item. Storage conditions were detailed on 93.1% of the products while allergen information was present on 66.6% of the products. Manufacturer’s details, date of manufacture and expiry were mentioned on all the products. Product labeling was compared with ‘Labeling requirements’ as suggested by Food Safety and Standards (Labeling and Display) Regulations, 2020 and is described in Table 4.

**Table 4:** Information available on the package of the millet-based products (*n* = 290).

Variables	N	(%)
Name of the product	290	(100)
Ingredient label present on the product	290	(100)
Declaration regarding Veg or Non-veg	290	(100)
Declaration regarding food additives/colors/flavors	266	(91.7)
Manufacturer’s details	290	(100)
Net quantity	290	(100)
Lot/Code/Batch identification	290	(100)
Date of manufacture and expiry	290	(100)
Country of origin	290	(100)
Instructions for use/cooking instructions		
Mentioned	176	(60.7)
Not mentioned	5	(1.7)
Not applicable (in some ready-to-eat products)	109	(37.6)
Language used for cooking instructions (*n* = 176)		
Only English	172	(97.7)
English and Hindi	3	(1.7)
English, Hindi and Tamil	1	(0.6)
Health claims	279	(96.2)
Added vitamins and minerals	219	(75.5)
Suitable age range	132	(45.5)
Storage conditions	203	(70)
Allergen info	196	(67.6)
FSSAI label	286	(98.6)
Cost of product	290	(100)

Contact information that indicated a customer’s right to provide feedback was present on 96.9% of the products. Around 95% of the products had the e-mail id mentioned followed by phone number (88.5%), website address (44.1%), postal address (35.1%) and toll-free number (3.2%) as a mode for feedback. Social media logo or id (WhatsApp, Facebook, Instagram, Pinterest, LinkedIn, YouTube, twitter) was mentioned in about 25% of the products. The various packaging materials used for the products included: paper (56.9%), plastic (38.3%) and glass (4.8%). Product packaging quality was assessed based on parameters like convenience, vulnerability to leakage or contamination. A majority of the products (96.2%) were found to be of good/ reasonable quality while 3.8% products were of average quality of packaging. Of the 290 products, 61.7% were of good/very good overall attractiveness (assessed subjectively using a 5-point Likert scale based on fonts and colors used, illustrations, striking taglines etc.) while around 29% products were rated as average and 9.3% products were rated poor/very poor.

The result from the face-to-face interview and market survey reveals that people consume mainly Ragi, followed by jowar and bajra, respectively. This is in concordance with them being mainly cultivated in the state of Telangana.

## 4 Discussion

The dietary practices related to millet-based foods among under-five children attending a tertiary care hospital in Telangana were assessed by interviewing the mothers of the selected children. Most of the interviewed mothers were homemakers, and also the primary caretakers of their children, and therefore were well-placed to detail the dietary practices of their children. Millets are an integral part of the traditional food practices in the state of Telangana, and unsurprisingly over 99% of the mothers were aware of millets, in particular Jowar, Ragi, and Bajra. These three millets constitute the major millets cultivated in Telangana, explaining this high level of awareness (23). However, incorporation of millets into routine dietary practices was found to be limited. Even though less than half of these mothers included millets in their child’s diet, a common trend of ragi followed by jowar and bajra, respectively, was seen in the consumption pattern as well as market profile. This is in line with the production pattern of the state. The commonly stated reasons for feeding children millets were the mothers’ perceptions that millets were healthy, strengthened bones and helped in the growth and development of children. These positive perceptions might be the result of an interplay between traditional wisdom and cultural factors, as family and friends were the major source of information regarding millets.

Among the children fed a millet-based diet, majority received it as a major meal, at least 2–3 times a week. Millets provide a multitude of health-related benefits to growing children; they possess a rich, diverse mix of macro and micronutrients. They also have growth promoting effects, strengthen bones and help in combating iron-deficiency anemia by improving hemoglobin levels in young children (18, 24–27). On the contrary, some of the common reasons for not including millets in their children’s diets were a lack of awareness that young children could be given millets and children’s dislike for the taste. Our findings are corroborated by other studies, which also report that a less desirable taste is one of the most common reasons for non-consumption of millets (8, 16, 28). However, in children consuming millets, over two-thirds liked the foods very much or to a good extent in our study. The taste of millets can be improved by techniques like soaking, germination, and fermentation, and the acceptance by children can be increased by utilizing millets in foods like muffins, cookies, and millet bars (16, 24).

According to the NFHS-5, 2019–21, the percentage of under-five children with severe wasting in Telangana stood at 9%, a lower figure compared to our study, wherein 14.1% were found to be suffering from SAM (3). This could be due to the fact that this study was conducted among under-five children attending a tertiary care hospital, whose characteristics may not be representative of the children residing in the broader community. A comparison of anthropometric characteristics based on the consumption of millet-based foods found that the height, weight and mid-upper arm circumference was significantly higher in children who were fed millets while the presence of MAM and SAM was noted to be significantly lower in the group of children who were fed millets. Although a causal association cannot be established due to the cross-sectional nature of the study, existing literature has shown that millet-based diets promote growth and play a beneficial role on the nutritional status of young children, improving their height, weight and MUAC across multiple contexts (25, 29). The mothers who provided millet might be aware of the nutrition and health status of their children, which was not explored in detailed in this study. The Government of India’s decision to mainstream millets in child nutrition through their introduction in the supplementary nutrition provided at the Anganwadis under the ICDS program is thus a welcome step toward combating childhood malnutrition (30). In addition, educational activities directed toward mothers to popularize the inclusion of millets in the diets of young children need to be undertaken to improve their acceptance and uptake. However, we should also acknowledge the fact that in spite of higher levels of iron, calcium, dietary fibers, etc., millets lack some essential nutrients like vitamin B12, lysine, etc., and are not a complete nutrition. Hence, promotion of millet consumption should be coupled with dietary diversity, since the inclusion of millet supports sustainable and acceptable eating habits to address malnutrition.

The packaging and labeling of the food items was assessed based on the regulations stipulated by the FSSAI (19). Full compliance was noted with respect to the name of the product, ingredient label, manufacturer details, dates of manufacture and expiry, and cost. The FSSAI also mandates that all food items should have symbol indicating whether they are vegetarian and non-vegetarian. This is particularly important in a country like India, where food consumption patterns are diverse and heavily influenced by cultural, social and religious factors. Most of the RTC food items had cooking instructions written on them, which were present in English on all of the items, and in Hindi and Tamil in very few. This ensured compliance with the FSSAI’s language regulations but the glaring absence of instructions in the vernacular language, Telugu, prevents a significant number of individuals from using the product as intended. This must be addressed so that the benefits of these products can be enjoyed by a wider segment of the local population. Moreover, more than half of the products lacked any information of the suitable age range for the food item. This is important as nutritional requirements vary across the different stages of life, and certain constituents might not be suitable for a particular age range especially for under-five children. Moreover, mothers interested in feeding their child millets may abstain from feeding their children such products due to an absence of information. Information regarding suitability of a food item for young children is therefore paramount to safeguard their health and ensure uncompromised nutrition. Information on saturated fats, trans fats and sugar, whose consumption must be strictly moderated was also missing in several products (31). Alarmingly, allergen-related information was found on just two-third of the products, which could have serious consequences. These shortcomings in labeling constitute a major health hazard to consumers and must be corrected with due haste through appropriate regulatory interventions.

The assessment also revealed that the packaging of most items was reasonable to good in quality and was attractive in a little over 60% of the products. Most products also bore a health claim promoting the products’ healthiness and superior nutritional qualities. Attractive packaging coupled with such health claims can strongly influence product choices among parents and even young children, setting the tone for food habits that extend beyond childhood. While attractive and informative packaging can help promote uptake of healthier foods, it could also lead to the popularization of nutritionally poor or inappropriate foods (32–34). The nutritional profile of the analyzed food revealed interesting insights upon comparison with the nutritional requirements of young children. Positively, 100 grams of a majority of the RTC and RTE items could meet up to half of the daily energy and at least half of the daily protein requirements. These commonly available items can therefore be easily integrated into diets to meet the macronutrient requirements of young children. The picture for micronutrients was however different, a 100-gram portion of the majority of these items could fulfill less than a quarter of the daily calcium requirements. In contrast, a majority of the products could meet more than three-quarters of the daily iron requirements in children aged 6–12 months but could meet only up to half of the requirements for higher age groups like 3–6 years. We also found that these items, particularly RTC items contained relatively high amounts of sodium. High sodium intake in childhood can increase the risk of hypertension, obesity and other non-communicable diseases in adulthood and it is therefore important to limit its consumption even among young children (35, 36). The regulations on the content of sodium in RTE and RTC needs to be strengthened as these products are widely consumed by all age groups across globe. These RTC and RTE items are a quick, convenient and appealing way of introducing millets to improve the dietary diversity and health of young children. However, due emphasis on parental awareness and education is necessary to ensure that they make informed choices while selecting items to ensure that micronutrient needs are optimally met. This assessment also makes a strong case for biofortification of these millet-based products, a proven and cost-effective strategy to alleviate micronutrient deficiencies (37). Though more than 40% of millet products available in the market are RTE type, only 4% of mothers included in the study reported that they give either RTE or RTC millet-based food for their children. The primary catchment area for the health facility included in the study is rural (urban/ rural residence data was not collected), and millet being a staple food, it is common practice in rural areas to make millet flour at home and use it. More than 90% of the mothers are educated at a high school level or above, and more than 90% are the primary caretakers of the children, and the median family income is INR 20,000, indicating that the education, income, or caregiver-related factors may not be the reason for less utilization of RTE/ RTC millet-based foods. The labeling information on the RTE/ RTC millet products was mainly in English and none in Telugu (Telugu is the regional language for the state of Telangana). This might have also limited the purchase of millet-based RTE/RTC products.

The present study is the first of its kind in India that delves into food practices related to millet-based food among mothers of young children. The nutritional evaluation of commonly available millet-based packaged foods is also a novel component of this study and covers a wide range of products across both online and offline platforms. However, the study has certain limitations. The cross-sectional nature of the study prevents us from determining the causality of the associations reported in the study. Moreover, the study population comprised of young children who reported to the center for healthcare, and may not be representative of the wider population in the community, limiting generalizability. The assessment of packaging quality and attractiveness may also have resulted in observer bias, as it was a subjective process. Evaluation of the nutritional profile of the packaged foods was based on every 100 g of the product. Social desirability bias could also be a concern as the study mainly relies on self-reported data by the mothers of these young children. With the increasing retail price of millets, the economic conditions of the family, the educational status of the mother and influence of other family members on child feeding practices might have played a role in millet product consumption among children. The awareness of the need for dietary diversity might be lacking in less educated mothers, and poor socioeconomic households might include cheaper staple dietary components as food, and hence neglect complete nutrition. Urban residence and employment status of parents can also act as potential confounders due to better access. However, the study also highlights a gap in the current food habits among children, emphasizing a need to integrate millet-based preparations into the nutrition-related programs targeted toward children in India (ICDS under POSHAN 2.0). This may further strengthen the POSHAN 2.0 on dietary diversification and life cycle-based nutrition in combating undernutrition, micronutrient deficiencies and the increasing epidemic of overweight among children in the country. Furthermore, the millet product profiling in the market has revealed gaps in availability and labeling of packaged millet products, suggesting the need for regulatory support from FSSAI.

## 5 Conclusion

This study highlights the dual potential of millet-based foods to enhance dietary diversity and address malnutrition in under-five children in India. While awareness of millets was high among mothers, actual incorporation into young children’s diets remains limited. Children consuming millets demonstrated significantly better anthropometric outcomes. However, the nutritional content and labeling of millet-based packaged products varied widely, with notable gaps in micronutrient content, high sodium and age-specific information. To maximize health benefits, efforts should focus on nutrition education for caregivers, fortification of products, and food product labeling regulations. Future studies on the direct effect of millets in combating childhood malnutrition need to be explored.

## Data Availability

The raw data supporting the conclusions of this article will be made available by the authors, without undue reservation.
